# Sudachitin, a polymethoxylated flavone, improves glucose and lipid metabolism by increasing mitochondrial biogenesis in skeletal muscle

**DOI:** 10.1186/1743-7075-11-32

**Published:** 2014-07-04

**Authors:** Rie Tsutsumi, Tomomi Yoshida, Yoshitaka Nii, Naoki Okahisa, Shinya Iwata, Masao Tsukayama, Rei Hashimoto, Yasuko Taniguchi, Hiroshi Sakaue, Toshio Hosaka, Emi Shuto, Tohru Sakai

**Affiliations:** 1 Department of Public Health and Applied and Nutrition, Institute of Health Bioscience, University of Tokushima, 3-18-15 Kuramoto, Tokushima 770-8503, Japan; 2 Tokushima Prefectural Industrial Technology Center, Tokushima, Japan; 3 Department of Metabolism and Nutrition, Institute of Health Bioscience, University of Tokushima, Tokushima, Japan; 4 Department of Endocrinology and Diabetes, Department of General Internal Medicine, School of Medicine, Saitama Medical University, Saitama, Japan

**Keywords:** Sudachitin, Glucose metabolism, Lipid metabolism, Mitochondria

## Abstract

**Background:**

Obesity is a major risk factor for insulin resistance, type 2 diabetes, and stroke. Flavonoids are effective antioxidants that protect against these chronic diseases. In this study, we evaluated the effects of sudachitin, a polymethoxylated flavonoid found in the skin of the *Citrus sudachi* fruit, on glucose, lipid, and energy metabolism in mice with high-fat diet-induced obesity and *db*/*db* diabetic mice. In our current study, we show that sudachitin improves metabolism and stimulates mitochondrial biogenesis, thereby increasing energy expenditure and reducing weight gain.

**Methods:**

C57BL/6 J mice fed a high-fat diet (40% fat) and *db*/*db* mice fed a normal diet were treated orally with 5 mg/kg sudachitin or vehicle for 12 weeks. Following treatment, oxygen expenditure was assessed using indirect calorimetry, while glucose tolerance, insulin sensitivity, and indices of dyslipidemia were assessed by serum biochemistry. Quantitative polymerase chain reaction was used to determine the effect of sudachitin on the transcription of key metabolism-regulating genes in the skeletal muscle, liver, and white and brown adipose tissues. Primary myocytes were also prepared to examine the signaling mechanisms targeted by sudachitin *in vitro*.

**Results:**

Sudachitin improved dyslipidemia, as evidenced by reduction in triglyceride and free fatty acid levels, and improved glucose tolerance and insulin resistance. It also enhanced energy expenditure and fatty acid β-oxidation by increasing mitochondrial biogenesis and function. The *in vitro* assay results suggest that sudachitin increased Sirt1 and PGC-1α expression in the skeletal muscle.

**Conclusions:**

Sudachitin may improve dyslipidemia and metabolic syndrome by improving energy metabolism. Furthermore, it also induces mitochondrial biogenesis to protect against metabolic disorders.

## Background

The prevalence of metabolic syndrome is increasing worldwide, and substantially increases the risk of metabolic and cardiovascular diseases [1]. Metabolic syndrome is characterized by various metabolic abnormalities, including central obesity, hyperlipidemia, hyperglycemia, and hypertension. These metabolic abnormalities may be caused by excess accumulation of visceral fat or abnormal accumulation of fat in non-adipose tissues, particularly the liver and skeletal muscle [2]. A sedentary lifestyle and high-fat Westernized diets have been implicated in the increasing prevalence of metabolic syndrome. Aside from lifestyle and diet, multiple metabolic pathways are implicated in the pathogenesis of metabolic disease, including aberrant lipogenesis, increased inflammation, and reduced fatty acid oxidation [3]. Therefore, in addition to identifying the underlying molecular mechanisms, and designing interventions to improve lifestyle and diet, it is also important to develop new medicines or identify new phytochemicals, such as flavonoids, that can improve these disorders.

Flavonoids are expected to have beneficial effects on metabolic activities, based on their hypocholesterolemic effects. Resveratrol is known to reduce fat accumulation and improve glucose tolerance and insulin sensitivity in mice with high-fat diet-induced obesity [4]. In addition to resveratrol, several polymethoxylated flavonoids (PMFs), including tangeretin, nobiletin, and naringenin, have been shown to exhibit metabolic effects [5,6]. Tangeretin was reported to decrease diacylglycerol acyltransferase (DGAT) activity, microsomal triglyceride accumulation, and apolipoprotein (apo) B100 secretion in HepG2 cells [6]. Naringenin was reported to inhibit lipogenesis and the secretion of very low-density lipoprotein–apoB and improve serum lipid levels in a mouse model of dyslipidemia and hyperinsulinemia [5,7]. Meanwhile, a mixture of tangeretin, nobiletin, and other PMFs was found to improve dyslipidemia and glucose intolerance by regulating adipokine secretion in hamsters with fructose-induced insulin resistance [8]. Nobiletin increased glucose transporter (GLUT)-4 expression in the muscle and decreased hyperglycemia in *ob/ob* mice [9]. Naringenin, resveratrol, and other polyphenols also suppressed arteriosclerosis via these metabolic effects in low-density lipoprotein receptor knockout and apolipoprotein E knockout mice [10,11]. Consequently, PMFs and related compounds were expected to have clinically useful anti-arteriosclerotic properties.

*Citrus sudachi* hort. ex. Shirai (Rutaceae), also known as sudachi, is an evergreen tree found primarily in the prefecture of Tokushima, Japan. Sudachi is a well-known citrus fruit in Japan, with approximately 8000 tons of fruit grown annually. Approximately half of the fruit is sold to be eaten, while the rest is squeezed and processed into juice. The residue from the fruit processing amounts to approximately 200 tons/year, and there is increasing demand to use it effectively. The flavonoids extracted from the peel of the sudachi fruit have been shown to exhibit biological activities, including antioxidant, antimicrobial, and anti-diabetic effects (unpublished data). The peel can also reduce triglyceride levels and improve obesity or diabetes (data not published). However, it is not known at this time which component of sudachi is responsible for these effects, or which signaling pathway is involved. In this study, we extracted the PMF sudachitin (5,7,4′-trihydroxy-6,8,3′-trimethoxyflavone) from the peel of *Citrus sudachi* and evaluated its anti-obesity and anti-diabetic effects in two distinct models of metabolic dysfunction. C57BL/6 mice fed a high-fat diet exhibit many features of metabolic syndrome including obesity, hyperinsulinemia, hyperlipidemia, and glucose intolerance [12]. *db/db* mice, a model of type 2 diabetes, is based on a leptin receptor mutation and shows hyperphagia, obesity, hyperglycemia, hyperinsulinemia, and hepatic steatosis [13,14].

Diabetic changes are generally considered to involve alterations in the anatomy, endocrine responsiveness, and metabolic function of the white and brown adipose tissues (WAT and BAT, respectively), liver and skeletal muscle. In adipose tissue, insulin resistance is manifested by reduced glucose uptake and increased free fatty acid release [15]. Adipokine dysfunction plays an important role in the development of obesity and insulin resistance [16,17]. The liver plays an important role in maintaining glucose homeostasis and controlling lipid metabolism [18], while skeletal muscle is a key tissue involved in glucose uptake. Peroxisome proliferator-activated receptor (PPAR)γ induces adipocyte differentiation and regulates the expression of several key genes involved in lipid and glucose metabolism [19]. Therefore, we examined the expression of PPARγ and its target genes.

We hypothesized that sudachitin will exhibit significant anti-obesity and anti-diabetic effects, as it is a tridemethyl form (4′, 5, 7) of nobiletin, and we evaluated its effect on metabolic parameters in animal models of obesity and diabetes. To elucidate the cellular mechanisms by which sudachitin exerts this effect, we evaluated the transcription of genes encoding regulators of glucose and lipid synthesis, trafficking, and responsiveness.

## Methods

### Microwave-assisted extraction of Sudachitin

The green peels of *Citrus sudachi* Hort. ex Shirai were collected in Tokushima Prefecture, Japan, in September. The dried peels were ground in a grinder. Microwave-assisted extraction was performed using a micro-reactor (2.45 GHz, 700 W; Shikoku Instrumentation Co., Ltd., Kagawa, Japan). A mixture of dried ground sudachi peels (100 g) in MeOH (600 mL) and water (200 mL) was stirred in a separable three-necked round-bottom flask (1 L) with a condenser, thermo-sensor, and mechanical stirrer. The mixture was irradiated under microwaves (MW) for 12 min under reflux with stirring. The reaction mixture was extracted with ethyl acetate and separated into the ethyl acetate layer and water-soluble layer. The ethyl acetate layer was dried with Na_2_SO_4_, and ethyl acetate was subsequently removed under reduced pressure. The resulting dark green residue was chromatographed on silica gel column (elution with *t*-BuOH/hexane/ethyl acetate = 0.5 : 5 : 1.2) to yield sudachitin as yellow needles, which was recrystallized from ethyl acetate to give sudachitin (0.092 g, melting point 238–240°C). A mixture of the water-soluble layer (280 mL), MeOH (280 mL), and concentrated HCl (240 mL) in the round-bottom flask was irradiated by MW for 7 min under reflux with stirring. The reaction mixture was neutralized with NaOH, and MeOH was removed under reduced pressure to give a brown residue, which was extracted with ethyl acetate and dried (Na_2_SO_4_). After removal of ethyl acetate, the resulting brown residue was chromatographed on silica gel column (elution with *t*-BuOH/hexane/ethyl acetate = 0.5 : 5 : 1.2) and further recrystallized from ethyl acetate to give sudachitin (0.376 g, melting point 238–240°C) as colorless needles. The purity of sudachitin was confirmed by HPLC to be above 95%. Sudachitin was dissolved in 0.2% dimethyl sulfoxide (DMSO).

### Animals and experiments

All work involving animals was performed in compliance with the Guide for the Care and Use of Laboratory Animals and protocols approved by the Institutional Animal Care and Use Committee at the University of Tokushima Graduate School (Tokushima, Japan).

C57BL/6 mice at 4 weeks of age were purchased from Japan SLC, Inc. (Shizuoka, Japan). Mice were housed in temperature- (23 ± 3°C) and humidity-controlled conditions with a 12-h light/dark cycle. Mice were given free access to water and either a control diet (n = 20; 14% of calories from fat; Oriental Yeast Co., Ltd., Tokyo, Japan) or a high-fat diet (n = 20; 40% of calories from fat; Oriental Yeast Co., Ltd.). Mice were allowed to adapt to these conditions for 1 week before starting the experimental protocol. During the experimental period, the mice were divided into two groups (n = 10) and orally administered either 5 mg/kg sudachitin dissolved in a 0.2% sodium carbonate solution or a corresponding volume of 0.2% DMSO solution mixed with 0.2% sodium carbonate daily for 12 weeks. Body weight and food intake of each mouse were measured weekly.

Ten *db/db* mice at 4 weeks of age were purchased from Charles River Japan, Inc. (Kanagawa, Japan). Mice were housed under temperature- (23 ± 3°C) and humidity-controlled conditions with a 12-h light/dark cycle. Mice were given free access to water and the control diet, as described above, and allowed to adapt to the conditions for 1 week before the beginning of the experimental protocol. Mice were divided into two groups (n = 5) and orally administered 5 mg/kg sudachitin or 0.2% DMSO, as above, daily for 12 weeks. The body weights and food intake of mice were measured once a week.

### Plasma and tissue collection

Before, during, and at the end of the experimental period, blood samples were taken from the tails of the mice fasted overnight for biochemical analyses. At the end of the study, mice were sacrificed by cervical dislocation after blood collection. Plasma samples were prepared by centrifugation at 3,000 rpm for 10 min at 4°C, and were stored at -80°C until analysis. Samples of the liver, gastrocnemius muscle, epididymal (visceral) and inguinal (subcutaneous) WAT, and BAT were collected, rinsed, weighed, frozen in liquid nitrogen, and stored at -80°C until analysis.

### Plasma biochemical analyses

Plasma non-esterified fatty acid (NEFA), triglyceride, and total cholesterol levels were measured using enzymatic commercial assay kits (NEFA C-Test, Triglyceride E-Test, and Cholesterol E-Test, respectively; Wako Pure Chemical Industries, Osaka, Japan). Plasma insulin, adiponectin, and leptin levels were measured by enzyme-linked immunosorbent assays (ELISA; Mouse Insulin ELISA Kit, Mouse/Rat High Molecular Weight Adiponectin ELISA Kit, and Mouse Leptin ELISA Kit; Shibayagi, Gunma, Japan).

### X-ray CT scan

Total body fat, subcutaneous fat, and visceral fat were measured in mice under isoflurane anesthesia using LaTheta X-ray CT scanner LCT-200 (Hitachi-Aloka Medical, Ltd., Tokyo, Japan). Data was analyzed using Visualization LaTheta software (Hitachi-Aloka Medical Ltd., Tokyo, Japan).

### Blood glucose, oral glucose tolerance tests (OGTT), and insulin tolerance tests (ITT)

Blood glucose levels were measured in tail vein blood using a glucometer (Arklay, Kyoto, Japan). For OGTT, blood glucose levels were measured 0, 30, 60, 90, and 120 min after an oral glucose load (1 g/kg) following an overnight fast. For ITT, blood glucose levels were measured 0, 30, 60, and 90 min after an intraperitoneal injection of regular human insulin (Novo Nordisk Pharma Inc., Tokyo, Japan). Insulin was administered to C57BL/6 J mice at 0.75 mU/g body weight and 3 mU/g to *db/db* mice, following a 6-h fast.

### Total RNA isolation

Total RNA was extracted from WAT and the gastrocnemius muscle using RNeasy Lipid Tissue and RNeasy Plus Universal Mini Kits (QIAGEN, Valencia, CA, USA) according to manufacturer’s instructions. Total RNA was extracted from skeletal muscle myocytes using RNAiso and amplified using CellAmp Whole Transcriptome Amplification Kit Version 2 (Takara Bio Inc., Shiga, Japan).

### Gene expression analyses

Total RNA (1 μg) was reverse-transcribed to cDNA in a final volume of 20 μL using the Primescript RT Reagent kit (Takara). Real-time polymerase chain reaction (PCR) was performed in a final volume of 10 μL containing 50 ng of the cDNA template and primers, using a StepOnePlus Real-Time PCR System (Life Technologies, Carlsbad, CA, USA). The primers used for PCR are listed in Additional file 1: Table S1. We evaluated the RNA levels of glucose transporters (Glut 1–4), adiponectin, PPARγ, adipocyte protein 2 (AP2), cluster of differentiation 36 (CD36, fatty acid translocase), uncoupling protein 1, 2, and 3 (UCP1, UCP2, and UCP3), and Sirt1 in tissue samples. In cultured skeletal muscle myocytes, we measured the transcription of genes involved in the synthesis of fatty acids (fatty acid synthase (FAS), acetyl-coA carboxylase (ACC1)), triglycerides (diglyceride acetyltransferase (DGAT1 and DGAT2)), or both (SREBP-1), as well as genes associated with hepatic cholesterol synthesis (farnesyl diphosphate synthase (FDS), squalene synthase (SS), 3-hydroxy-3-methyl-glutaryl-CoA reductase (HMG-R), and synthase (HMG-S)), cholesterol sensitivity (microsomal triglyceride transfer protein (MTTP), low-density lipoprotein receptor (LDLR)), lipid oxidation (UCP2, acyl-coenzyme A oxidase (ACOX), PPARγ coactivator 1α (PGC1α)), lipolysis (hormone-sensitive lipase (HSL), carnitine palmitoyl transferase-α (CPT1α), adipose triglyceride lipase (ATGL)), and glycogenolysis (glucose 6-phosphatase (G6Pase)), phosphoenolypyruvate carboxykinase (PEPCK)). Evaluation of mitochondrial biogenesis in primary myocytes involved measurement of nuclear respiratory factor 1 and 2 (NRF1 and NRF2) and mitochondrial transcription factor A (mtTFA).

### Measurement of adipocyte size

Subcutaneous and visceral adipose tissue samples were fixed in 2% osmium tetroxide and shaken at 37°C for 7 days. The fixed tissues were filtered through a 250-μm and 25-μm mesh to obtain a single cell suspension. The diameter and distribution of adipocytes were measured using a Coulter Multisizer 3 particle counter (Beckman Coulter, Inc., Brea, CA, USA).

### Analysis of visceral adiposity

The volume and distribution of visceral fat at 4 weeks after starting sudachitin administration were evaluated by X-ray computed tomography (CT; LaTheta, LCT-200, Aloka, Tokyo, Japan) of the region between the first lumbar vertebra and the pubic bone, under isoflurane anesthesia. Data were analyzed from continuous 2-mm slice images (for quantitative assessment) using LaTheta software.

### Immunoblotting

Total protein was extracted as described previously [20]. Lysates were separated by SDS-PAGE on 10% polyacrylamide precast gels (Invitrogen, Carlsbad, CA, USA) and transferred to polyvinylidene difluoride membranes by electroelution. Membranes were blocked in 20 mmol/L of TBS solution with 1% Tween containing 5% skim milk and incubated with primary antibodies overnight at 4°C. Polyclonal antibody sensitive to AMPKα1 and phospho-AMPKα1 (Thr172) was obtained from Cell Signaling Technology (Danvers, MA, USA) and antibodies against GLUT4 and β-tubulin were obtained from Santa Cruz Biotechnology (Santa Cruz, CA, USA). Protein density were visualized on membranes using horseradish peroxidase (HRP)-conjugated secondary antibodies (Santa Cruz Biotechnology) and visualized using the enhanced chemiluminescence reagent (GE Healthcare; Waukesha, WI, USA), according to manufacturer’s directions.

### Indirect calorimetry

Four-week-old male C57BL/6 J mice were fed high-fat diet and treated with sudachitin (5 mg/kg) or vehicle for 4 weeks. Oxygen consumption was continuously measured during the 12-h light–dark cycles using a comprehensive laboratory animal open-circuit indirect calorimetry monitoring system (Columbus Instruments, Columbus, OH, USA). Data were corrected for 2 days after 2 days of adaptation to the metabolic cages.

### Skeletal muscle enzyme activities

Skeletal muscle samples were homogenized with extraction buffer (0.1 mol/L KH_2_PO_4_, 0.1 mol/L NaPHO_4_, and 2 mmol/L EDTA, pH 7.2). Citrate synthase activity was determined using a Citrate Synthase Assay Kit (Sigma-Aldrich, St. Louis, MO, USA).

### ATP content measurement

ATP content was measured using the AMERIC-ATP kit (Applied Medical Enzyme Research Institute Corporation, Tokushima. Japan) according to manufacturer’s instructions. Briefly, a sample of skeletal muscle (100 mg) tissue was homogenized in 10× volume of extraction buffer (0.25 mol/L sucrose, 10 mmol/L HEPES-NaOH, pH 7.4) and centrifuged at 1000 g for 10 min. The supernatant was diluted 8-fold in phenol-based extraction buffer and ATP extraction reagent was added. Following addition of luciferase solution, ATP content was quantified by measuring the luminescence using a luminometer (Lumat LB9507, Berthold Technologies, Tokyo, Japan).

### Cell culture

Primary myoblasts were isolated from the calf region, thigh, and pelvic girdle of 8-week-old C57BL/6 J mice. Satellite cells were isolated by the digestion of muscle tissue by the type 2 collagenase (Worthington Biochemicals, Lakewood, NJ, USA), followed by pre-plating to purify cells and remove fibroblasts. Experiments were conducted after myoblasts had reached 99% purity. Cells were cultured at 37°C in 5% CO_2_ in Dulbecco modified Eagle medium (DMEM), supplanted with 10% fetal bovine serum (FBS) and 1% penicillin/streptomycin. Cells were differentiated in DMEM/20% horse serum supplanted with basic fibroblast growth factor for 3 days. The primary cells were treated with vehicle (0.2% DMSO) or 30 mmol/L sudachitin for 48 h.

### Mitochondrial staining

Skeletal muscle myocytes were grown and differentiated on a glass coverslip coated with Cell-Tak (BD Bioscience, Franklin Lakes, NJ, USA). The myocytes were stained with Mito-tracker dye (Molecular Probes, Invitrogen, Carlsbad, CA, USA) for 30 min and mitochondrial density was determined using optical microscopy (Eclipse TE 2000-U; Nikon, Tokyo, Japan). The density of mitochondria was quantified using Image J software (NIH, Bethesda, MD, USA).

### Statistical analysis

Data were expressed as means ± standard deviation (SD). Unpaired Student’s *t* test was used to statistically compare vehicle- and sudachitin-treated groups. Statistical significance was set *a priori* at *P* < 0.05. All analyses were performed using GraphPad Prism version 5.0 (GraphPad Software, San Diego, CA, USA).

## Results

### Sudachitin treatment prevents high-fat diet-induced weight gain, and reduces fat content and adipocyte size

**Figure 1:**
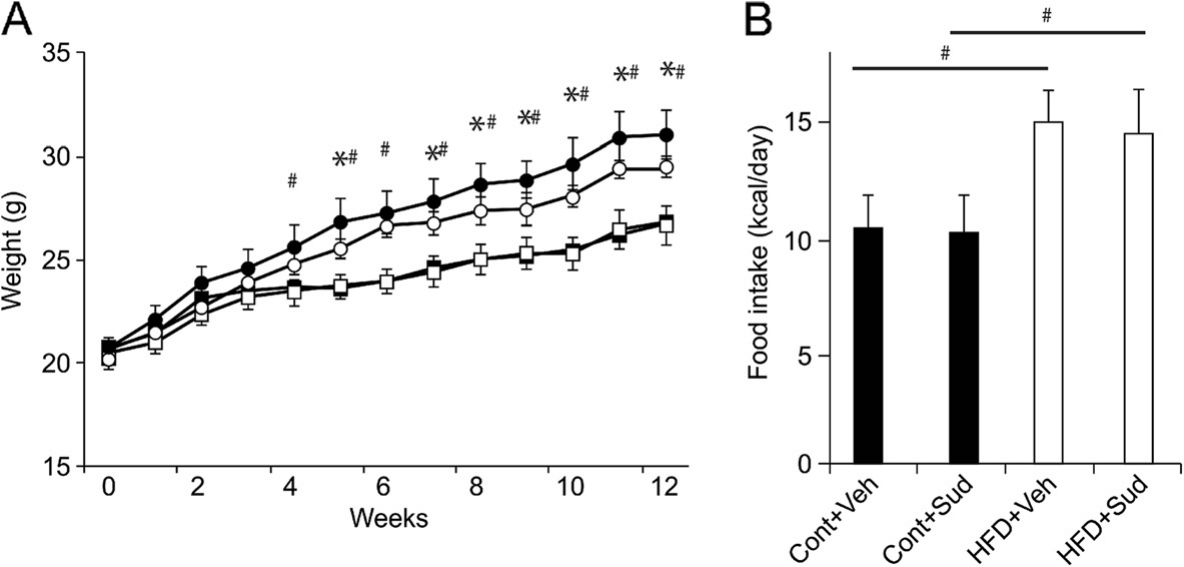
**Sudachitin inhibits weight gain in high-fat diet-fed mice. Mice were fed a low-fat control diet or a high-fat diet and were treated with 5 mg/kg sudachitin or vehicle for 12 weeks (n = 10 per group).** Mice were fasted for 16 h at the end of the study. Body weight was measured weekly **(A)** and food intake was measured every 2 days **(B)** for 12 weeks. Values are mean ± standard error of the mean (A) or mean ± standard deviation (B). *P < 0.05 sudachitin administration vs. control treatment, # *P* < 0.05 high-fat diet-fed vs. control diet-fed animals. Cont + Veh: vehicle-treated, control diet group (closed squares in panel A); Cont + Sud: sudachitin-treated, control diet group (open squares); HFD + Veh: vehicle-treated, high-fat diet group (closed circles); HFD + Sud: sudachitin-treated, high-fat diet group (open circles).

**Figure 2:**
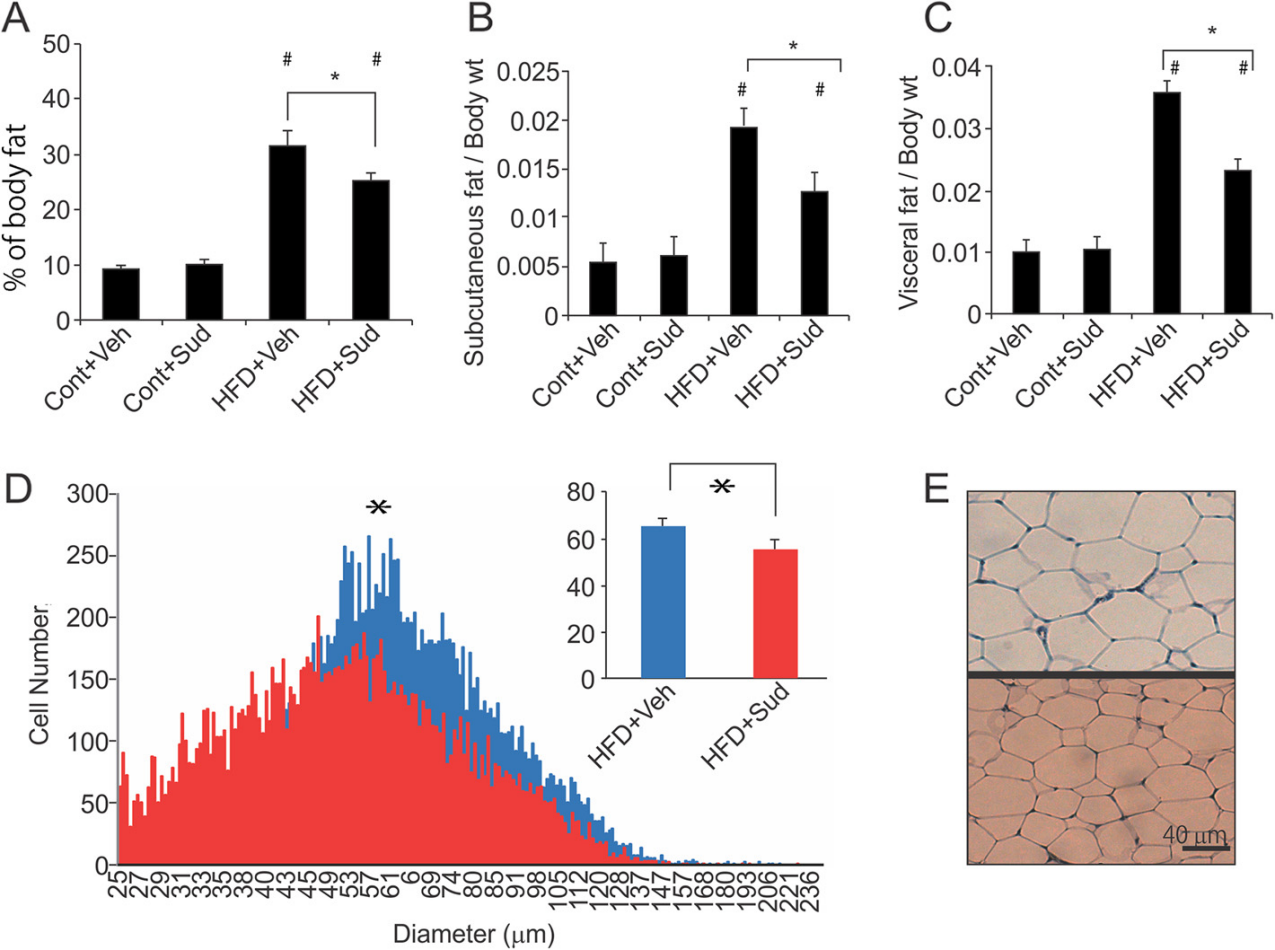
**Sudachitin reduces fat in high-fat diet-fed mice. (A)** Body composition was assessed by computed tomography, with the relative body fat content, subcutaneous fat content (g/body weight; **B)** and visceral fat content (g/body weight; **C)** quantified. Adipocyte size was measured after 12 weeks of treatment with sudachitin or vehicle **(D)**. Hematoxylin/eosin staining of WAT after 12 weeks of feeding in vehicle- (upper panel) and sudachitin-treated (lower panel) mice **(E)**. Values are mean ± standard deviation (A-C). *P < 0.05 sudachitin administration vs. control treatment, # *P* < 0.05 high fat diet vs control diet. Cont + Veh: vehicle-treated, control diet group (closed squares in panel A); Cont + Sud: sudachitin-treated, control diet group (open squares); HFD + Veh: vehicle-treated, high-fat diet group (closed circles); HFD + Sud: sudachitin-treated, high-fat diet group (open circles).

**Figure 3:**
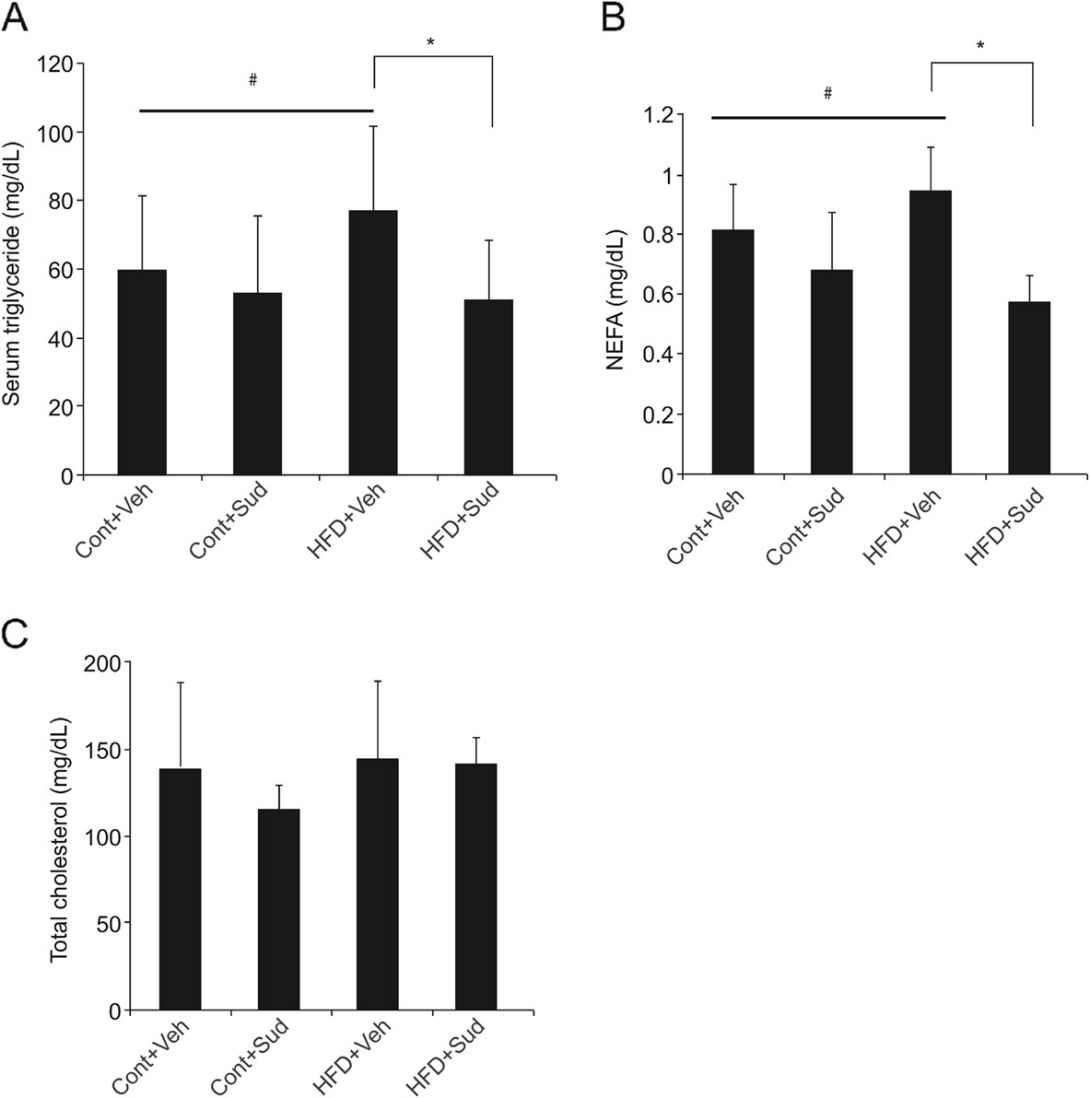
**Sudachitin reduces serum triglyceride and non-esterified fatty acid.** Serum triglyceride **(A)**, non-esterified fatty acid **(B)**, and total cholesterol (C levels were evaluated at the end of the 12-week treatment. Values are means ± standard deviation **(A-C)**. *P < 0.05 sudachitin administration vs. control treatment, # *P* < 0.05 high-fat diet-fed vs control diet-fed animals. Cont + Veh: vehicle-treated, control diet group (closed squares in panel A); Cont + Sud: sudachitin-treated, control diet group (open squares); HFD + Veh: vehicle-treated, high-fat diet group (closed circles); HFD + Sud: sudachitin-treated, high-fat diet group (open circles); NEFA: non-esterified fatty acid; T-CHO: total cholesterol.

As shown in Figure 1A and B, treatment with 5 mg/kg body weight sudachitin reduced the weight gain in mice fed a high-fat diet, without affecting the food intake. By contrast, treatment with sudachitin did not affect the body weight in mice fed a low-fat control diet. Treatment of mice fed a high-fat diet with 2 mg/kg sudachitin did not affect the body weight of the animals (data not shown). The magnitude of weight reduction was similar between 5 and 10 mg/kg doses of sudachitin (data not shown), which prompted us to use the 5 mg/kg dose for subsequent investigations.A high-fat diet resulted in significantly elevated total body adipose tissue, increased subcutaneous fat deposits and elevated visceral fat (enlarged epididymal fat pads) which were ameliorated by 5 mg/kg sudachitin administration (Figure 2A–C). However, there were no differences in BAT or muscle weight per body weight among the four groups (data not shown). Sudachitin treatment also significantly reduced adipocyte size in high-fat diet-fed mice, as compared to the vehicle-treated mice fed high-fat diet (Figure 2D and E). Since these changes were independent of food intake and muscle mass, the results suggest that sudachitin improves energy metabolism.We next examined the effects of sudachitin on metabolic parameters. As expected, a high-fat diet significantly increased serum triglyceride and NEFA levels by 1.5- and 1.3-fold, respectively, compared with the control diet (Figure 3A and B). Administration of 5 mg/kg sudachitin prevented these increases in triglyceride and NEFA levels. Total cholesterol levels were not significantly different among any of the studied groups (Figure 3C).

### Sudachitin improves glucose tolerance and insulin sensitivity in high-fat diet-fed mice

**Figure 4:**
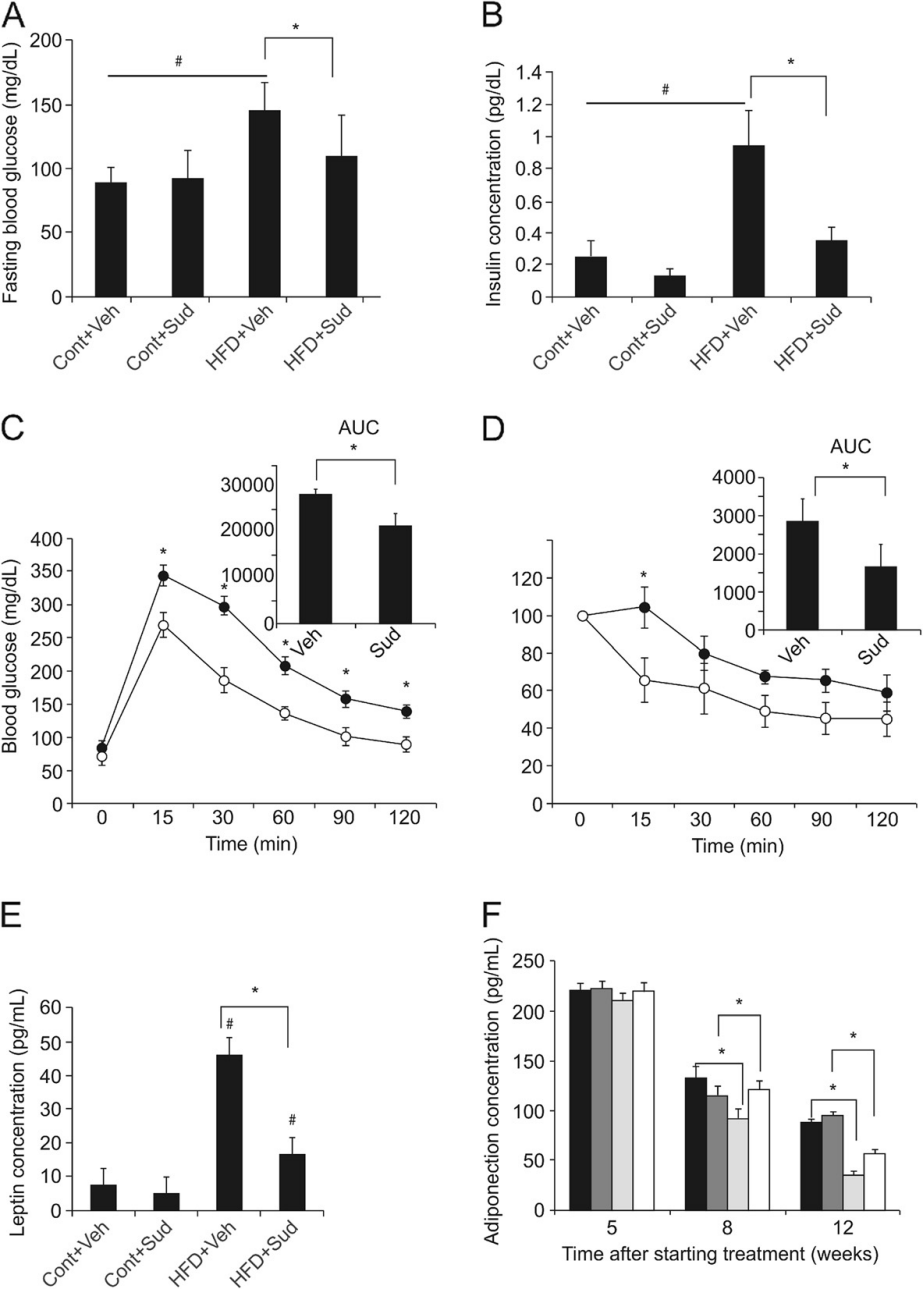
**Sudachitin improves glucose and insulin sensitivity in mice fed a high-fat diet.** Fasting blood glucose **(A)** and plasma insulin concentrations **(B)** after 12 weeks of sudachitin treatment. Glucose **(C)** and insulin **(D)** tolerance tests were performed in high-fat diet-fed mice after an overnight (16 h) **(C)** or 6 h **(D)** fast. Mice received an oral dose of 1 g/kg glucose **(C)** or 0.75 mU/kg insulin by intraperitoneal injection **(D)**. Blood glucose was measured at the indicated times. Glucose utilization and insulin sensitivity were determined from the area under the curve (AUC; inset). Open symbols: sudachitin-treated group; closed symbols: vehicle-treated groups. Plasma leptin levels were measured after 12 weeks of treatment **(E)**. Plasma adiponectin levels were measured after 5, 8, and 12 weeks of starting treatment **(F)**. Values are means ± standard deviation. Closed bars: vehicle-treated, control diet group; dark gray bars: sudachitin-treated, control diet group; light gray bars: vehicle-treated, high-fat diet group; open bars: sudachitin-treated, high-fat diet group; *P < 0.05 sudachitin administration vs. control treatment, # P < 0.05 high fat diet vs control diet-fed animals.

As shown in Figure 4A and B, mice fed a high-fat diet exhibited a state of moderate hyperinsulinemia and hyperglycemia, as compared to mice fed the control diet. Fasting plasma glucose and insulin levels were significantly reduced by sudachitin. Results of the OGTTs revealed that sudachitin improved glucose tolerance in high-fat diet-fed mice (Figure 4C). Sudachitin treatment also re-established normal insulin tolerance in high-fat diet-fed mice, normalizing the area under the glucose plasma concentration curve in these mice (Figure 4D). Mice fed a high-fat diet also exhibited elevated plasma leptin levels, which were ameliorated by sudachitin (Figure 4E), consistent with the observed sudachitin-induced decrease in visceral fat (Figure 2). Plasma adiponectin levels were found to decrease with age and to be significantly lower in mice fed a high-fat diet (Figure 4F). Sudachitin treatment increased plasma adiponectin levels in mice fed a high-fat diet but not in mice fed a control diet.

### Sudachitin alters the expression of metabolism-related genes in WAT and liver

**Figure 5:**
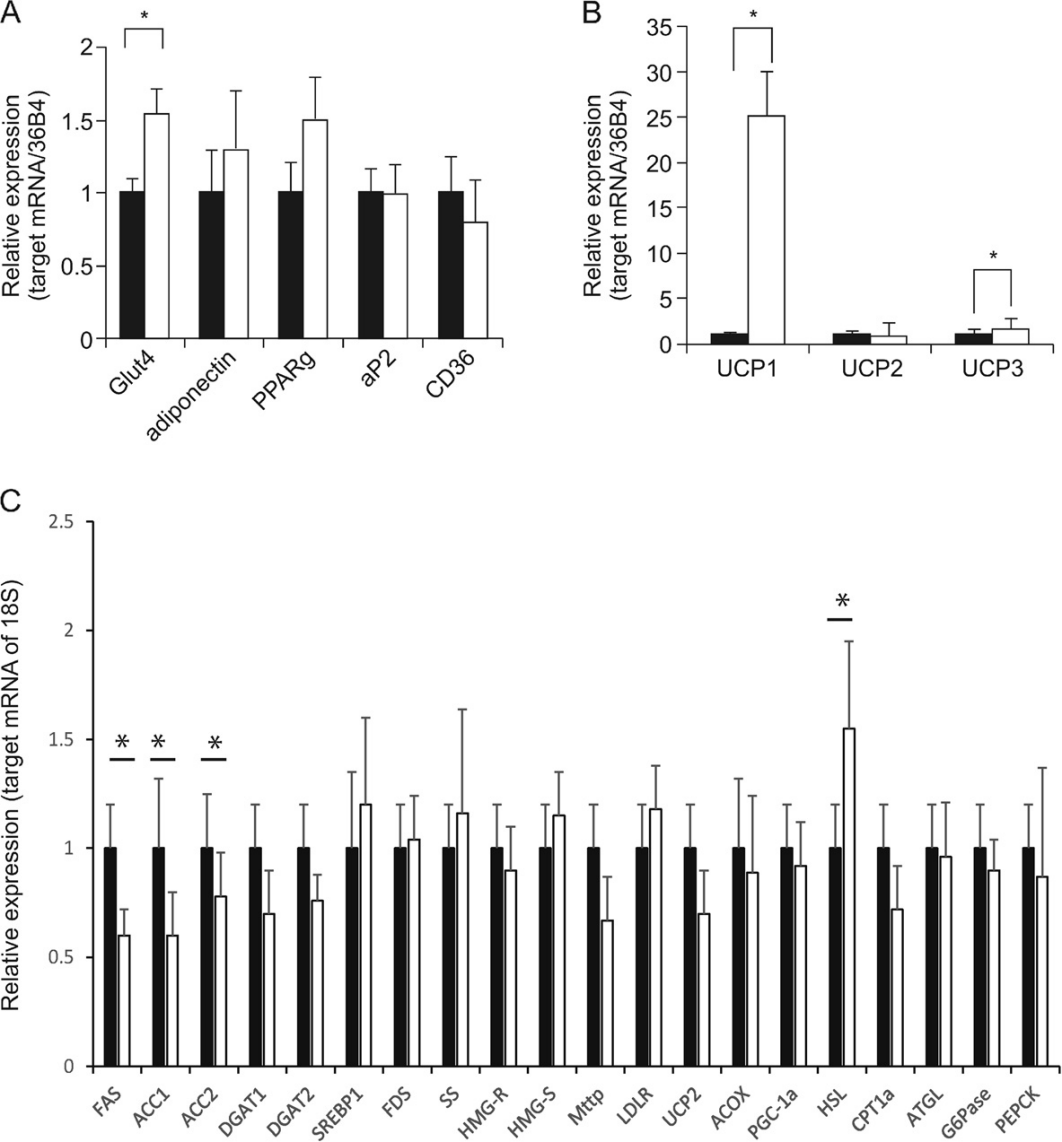
**Effects of sudachitin on mRNA levels in white adipose tissue and liver.** Gene transcription was normalized for 36B4 in subcutaneous white adipose tissue **(A-B)** or 18*S* in liver **(C)**. Data are means ± standard deviation. *P < 0.05 vs the indicated groups. Closed bars: vehicle-treated, high-fat diet group; open bars: sudachitin-treated, high-fat diet group.

As shown in Figure 5A, GLUT4 mRNA levels were significantly increased by the sudachitin treatment, suggesting a possible molecular mechanism underlying sudachitin-mediated improvement in glucose uptake. Transcription of the adiponectin gene in high-fat diet-fed mice treated with sudachitin showed a trend toward an increase that did not reach statistical significance, compared to the observation in vehicle-treated high-fat diet-fed mice. The mRNA level of PPARγ showed a trend towards an increase (1.5-fold) which was not statistically significant in the sudachitin-treated group compared with the vehicle-treated group. No significant differences in mRNA transcripts of adipocyte fatty acid-binding protein and CD36 were observed between the two treatment groups (Figure 5A). mRNA transcripts of uncoupling protein 1 and 3 (UCP1 and UCP3) were significantly increased in sudachitin-treated mice, which suggests that WAT gained a BAT-like phenotype, possibly resulting in increased thermogenesis.In the liver, we found that sudachitin decreased the levels of mRNA transcripts encoding FAS, ACC1 and ACC2, while the expression levels of DGAT1, DGAT2, and SREBP1, were not significantly different between vehicle- and sudachitin-treated mice fed a high-fat diet (Figure 5C). In addition, the expression levels of FDS, SS, HMG-R, and HMG-S were not affected by sudachitin (Figure 5C). The expression levels of MTTP and LDLR were not significantly different, although MTTP expression tended to be lower in the sudachitin-treated mice fed a high-fat diet as compared to the untreated high-fat diet-fed mice, although not significantly. There were no differences in the transcription of UCP2, ACOX, or PGC-1α between mice treated with sudachitin or vehicle (Figure 5C). However, transcription of the lipolytic gene HSL was increased in sudachitin-treated mice fed a high-fat diet, as was CPT1α, but there was no change in ATL (Figure 5C). The hepatic transcription of G6Pase and PEPCK, PPARγ, and adiponectin were not increased by sudachitin.

### Sudachitin improved insulin sensitivity in diabetic *db/db* mice

**Figure 6:**
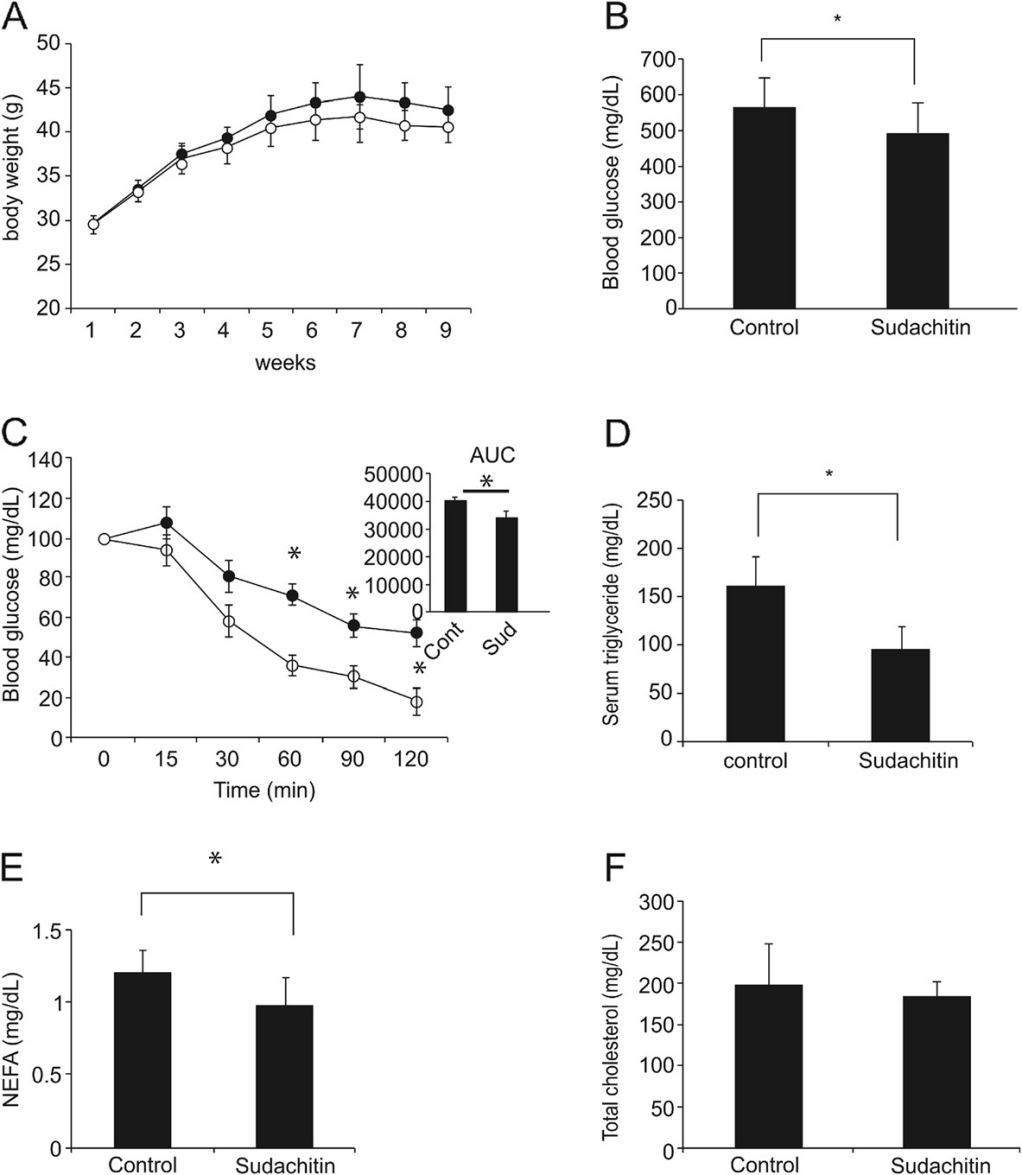
**Sudachitin improves fasting glucose, insulin sensitivity, and lipid metabolism in *db/db *mice.** While sudachitin treatment did not affect weight gain over 9 weeks of treatment **(A)**, it significantly decreased fasting blood glucose levels **(B)**. Insulin tolerance tests were performed after a 6 h fast **(C)**. Mice received an intraperitoneal injection of 3 mU/kg insulin, and blood glucose levels were measured at indicated times. Serum triglyceride **(D)**, non-esterified fatty acids **(E)**, and total cholesterol levels **(F)** were measured in *db*/*db* mice. Data are presented as means ± standard deviation. *P < 0.05 vs the indicated groups. closed circles: vehicle-treated group, open circles: sudachitin-treated group.

As shown in Figure 6A, body weight gain over the first 6 weeks of treatment in *db/db* mice was not significantly suppressed by treatment with 5 mg/kg sudachitin compared with vehicle-treated mice. All *db/db* mice were hyperglycemic when the experiment began, as confirmed by the average fasting blood glucose levels of 544 ± 96.0 mg/dL. Six weeks of treatment with sudachitin reduced fasting blood glucose levels compared with the untreated *db/db* mice (Figure 6B). Sudachitin also improved insulin sensitivity, as determined by the ITTs performed at this time (Figure 6C). As shown in Figure 6D–F, sudachitin significantly decreased serum triglyceride and NEFA, levels, but did not alter the total cholesterol levels.

### Sudachitin promotes energy expenditure by activating the Sirt1–PGC1α pathway

**Figure 7:**
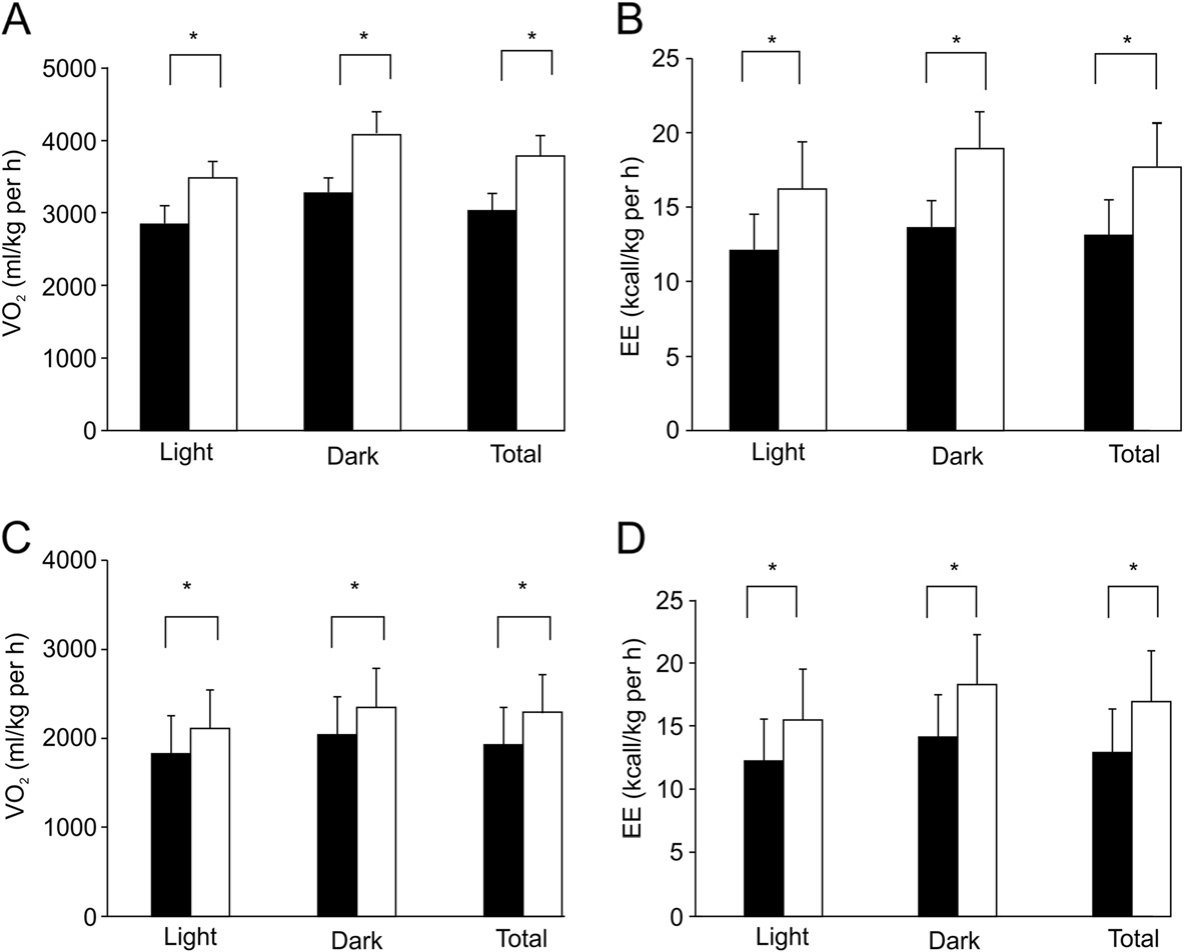
**Sudachitin increases energy expenditure.** Ten-week-old mice treated with vehicle or sudachitin (5 mg/kg) for 4 weeks were placed into an Oxymax open-circuit indirect calorimetry system for 4 days. Measurements of O_2_ consumption in control C57/Bl6 mice **(A)** or db/db mice **(C)**, and energy expenditure were obtained in control C57/Bl6 mice **(B)** or db/db mice **(D)**.

**Figure 8:**
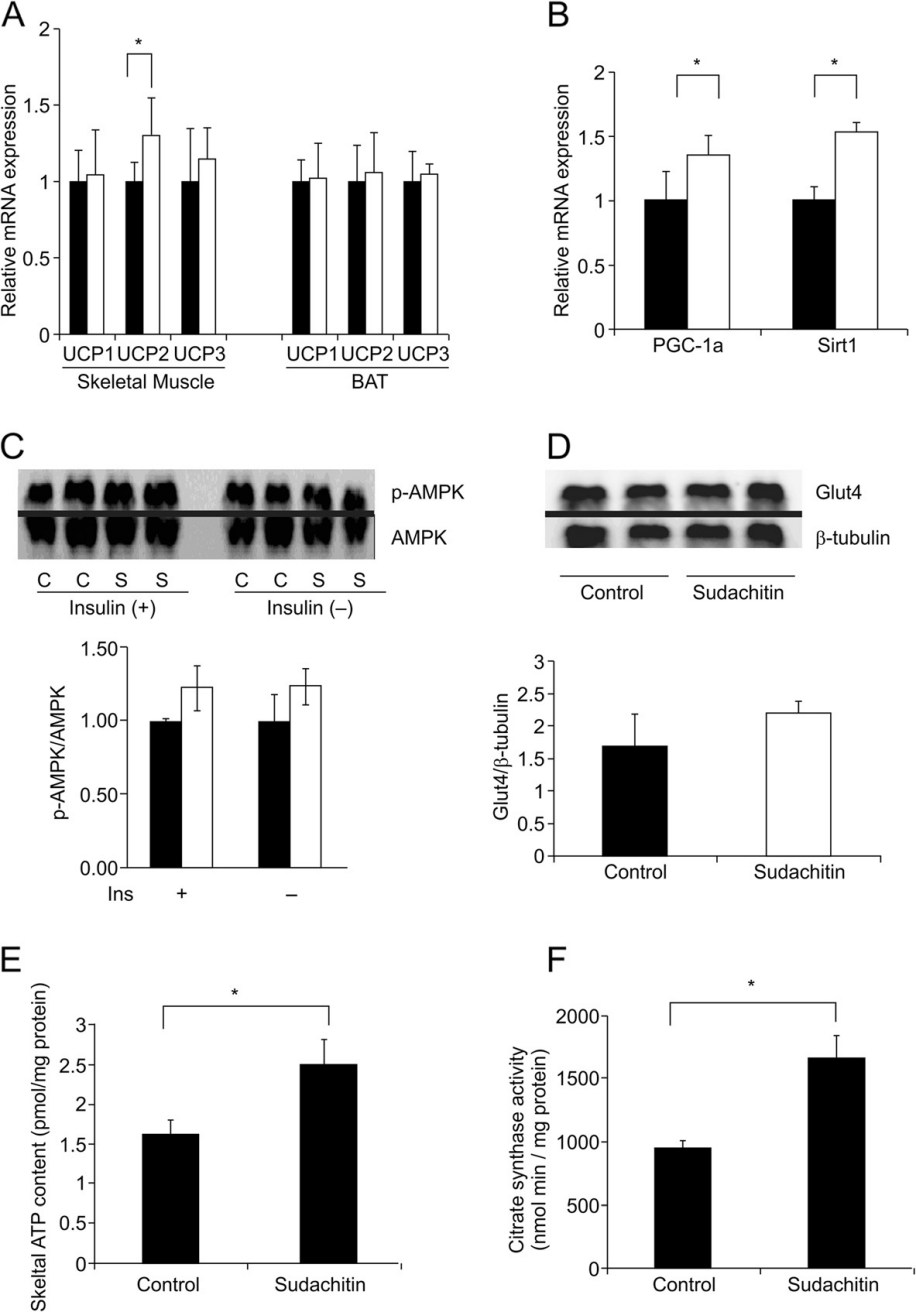
**Sudachitin increases energy metabolism-related gene expressions by activating mitochondrial biogenesis.** Relative mRNA expression of energy metabolism-related genes in the gastrocnemius muscle **(A, B)** and brown adipose tissue **(A)**. Gene expression was normalized for GAPDH in both tissues. Phosphorylation of AMPK in sudachitin- or vehicle-treated mice with or without insulin **(C)**. Protein expression of GLUT4 in mice administrated vehicle or sudachitin **(D)**. Skeletal muscle ATP content in mice fed a high-fat diet and treated with sudachitin or vehicle **(E)**. Citrate synthase activity was measured in mitochondria isolated from high-fat diet-fed mice treated with 5 mg/kg sudachitin or vehicle **(F)**. Data are presented as mean ± standard deviation. *P < 0.05 vs. the indicated group. Closed bars; vehicle-treated group; open bars: sudachitin-treated group.

Because sudachitin increased adiponectin levels and reduced adiposity without reducing food intake, we hypothesized that sudachitin increases the energy expenditure. To assess whole-body energy expenditure, mice fed a high-fat diet were treated with sudachitin or vehicle and subjected to indirect calorimetry. Oxygen consumption during the light and dark cycles was increased by the administration of sudachitin for 4 weeks, without weight change compared with control mice, resulting in an increase in total daily energy expenditure by 45% compared with vehicle-treated mice (Figure 7A and B). We observed similar increases in energy expenditure in *db/db* mice after 4 weeks of administration of 5 mg/kg per day of sudachitin (Figure 7C-D).Interestingly, in mice fed a high-fat diet, the respiratory quotient was not markedly different between mice treated without or with sudachitin (0.87 ± 0.04 and 0.83 ± 0.03, respectively). Because energy expenditure can be increased by mitochondrial uncoupling, we examined the expression of UCP1–3 in the gastrocnemius muscle and in BAT (Figure 8A). There were no significant differences in UCP3 expression levels in any of the tissues, although its expression was slightly increased in the skeletal muscle of sudachitin-treated mice. By contrast, UCP2 gene expression was significantly increased in skeletal muscle of sudachitin-treated mice. Sudachitin also induced the expression of PGC-1α and its target gene Sirt1 in skeletal muscle, which are known to regulate energy metabolism (Figure 8B). As shown in Figures 8C and D, there was a statistically insignificant trend toward increased phosphorylation of AMP protein kinase (AMPK) and Glut4 expression in response to sudachitin treatment. We also observed a 1.3-fold increase in basal ATP content (Figure 8E), as well as increased citrate synthase activity (Figure 8F) in sudachitin-treated mice, consistent with enhanced mitochondrial content or activity.

### Sudachitin promotes mitochondrial biogenesis by activating its signaling pathway in myocytes *in vitro*

**Figure 9:**
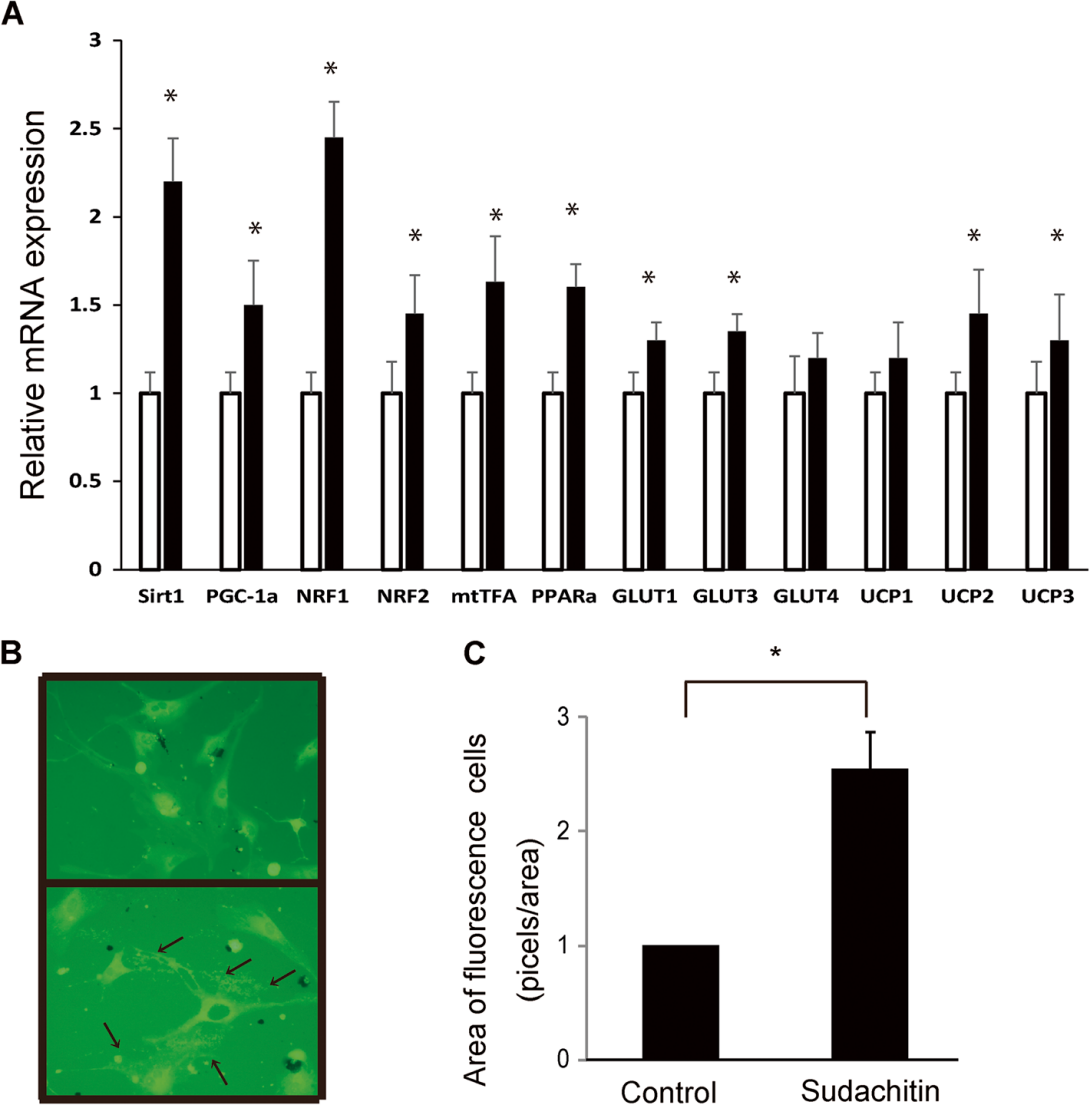
**Effects of sudachitin on gene expression in mouse primary skeletal muscle myocytes *in vitro*.** Gene transcription in myocytes treated with 30 μmol/L sudachitin or vehicle for 48 h, normalized for β-actin **(A)**. Data are means ± standard deviation. *P < 0.05 vs the indicated group. Closed bars: untreated cells; open bars: sudachitin-treated cells. The number of mitochondria in myocytes was increased by sudachitin **(B, C)**. Myocytes were incubated with the fluorophore Mito-Tracker Green, which specifically labels mitochondria. Upper panel: control cells; lower panel: cells treated with 30 μmol/L sudachitin for 48 h. Arrows indicate stained mitochondria **(B)**. Quantitative analysis of mitochondrial staining. Fluorescence intensity was measured by ImageJ software using the analyze particle function. *P < 0.05.

To determine the direct effects of sudachitin on muscle metabolism, we assessed the expression of key genes and mitochondrial number *in vitro* in primary cultured myocytes following incubation with sudachitin. Consistent with the observed increase in PGC-1α and Sirt1 mRNA in mice (Figure 8), transcription of these genes was significantly increased in differentiated myocytes exposed to 30 μmol/L sudachitin compared with the vehicle-treated cells (Figure 9A). Considering the genes involved in mitochondrial biogenesis, the expression of NRF1, NRF2, and mtTFA were increased by 1.5- to 2.5-fold following sudachitin treatment. In addition, the expression of UCP2 was significantly increased and UCP3 expression was slightly increased in myocytes treated with sudachitin for 48 h, whereas UCP1 was not different between control and sudachitin-treated myocytes. Because GLUT4 expression was increased in sudachitin-treated mice, we also determined the expressions of GLUT1, 3, and 4. As shown in Figure 9A, only the insulin insensitive transporters GLUT1 and 3 were significantly increased by sudachitin, whereas GLUT4 expression was not affected. To examine the effects of sudachitin on mitochondrial number and activity, we stained the mitochondria of myocytes and present representative images in Figure 9B and C, with stained mitochondria indicated with arrows. Sudachitin treatment increased mitochondrial staining with the fluorophore Mito-Tracker Green in skeletal muscle myocytes approximately 2.5-fold, in comparison to the cells treated with vehicle.

## Discussion

The present study revealed that sudachitin, a PMF extracted from the peel of the *Citrus sudachi* fruit, exhibits positive effects on glucose and energy metabolism in two animal models of metabolic disorders. Previous studies have shown that dietary supplementation with a mixture of PMFs nobiletin and tangeretin restores glucose homeostasis in hamsters with fructose-induced insulin resistance [21]. Several reports have demonstrated that citrus flavonoids, including hesperidin, naringin, and nobiletin have hypoglycemic effects [9,22]. In the present study, we found that plasma glucose and triglyceride levels in C57BL/6 J mice fed a high-fat diet were significantly reduced by treatment with 5 mg/kg sudachitin within 5 weeks of starting treatment. However, these reductions were not observed in mice treated with 2 mg/kg body weight sudachitin. Because the reductions in plasma glucose and triglyceride levels were similar between mice treated with 5 and 10 mg/kg body weight sudachitin, we used the 5 mg/kg dose in this study. This dose seems very low compared with that required for other flavonoids or other treatments. In experiments using pharmacological agents, it is important to use the lowest effective dose to minimize the chances of off target/side effects. We propose that the observed reduction in weight gain observed in sudachitin-treated mice is a result of altered glucose and lipid metabolism, caused by the changes in energy expenditure. This mechanism is supported by the sudachitin-mediated enhanced glucose metabolism observed in *db*/*db* mice, even without any weight loss.

How does sudachitin act on skeletal muscle in mice? So far, the mechanisms involved in the lipid-lowering effects of citrus flavonoids have not yet been fully established. In general, flavonoids are conjugated in blood, although it is possible that effects in certain tissues are mediated following local deconjugation. Nevertheless, we found that mice treated with sudachitin showed increased skeletal muscle mitochondrial biogenesis and function, and possibly increased NAD^+^ levels in skeletal muscle. Genetic disorders associated with impaired mitochondrial function are characterized by a rapid onset of symptoms that are commonly observed in elderly individuals, including clinical symptoms of type 2 diabetes [23]. There is increasing evidence that a progressive decline in mitochondrial function in asymptomatic, apparently healthy individuals may underlie a number of late-onset age-related diseases [24,25], and that treatments that stimulate mitochondrial function can delay the progression or even prevent the manifestation of some of these diseases [4,26,27]. Skeletal muscle is one of the primary tissues responsible for insulin-stimulated glucose uptake, and reduced mitochondrial function plays an important role in the development of insulin resistance coupled with obesity [23,28]. Therefore, activating upstream pathways that regulate mitochondrial biogenesis may prove effective in delaying and treating a variety of metabolic diseases.

PGC-1α is a transcriptional coactivator that regulates genes involved in energy metabolism and provides a direct link between external physiologic circuits and the regulation of mitochondrial biogenesis [29]. PGC-1α is expressed at high levels in tissues characterized by abundant mitochondria and active oxidative metabolism, such as BAT, the heart, and skeletal muscle, whereas its expression is much lower in the liver and WAT [30]. In this study, we found an increase in PGC-1α transcription in skeletal muscle, but not in BAT, following sudachitin treatment, suggesting that sudachitin, or its metabolite, act directly on the skeletal muscle. PGC-1α induces the transcription of NRF1 and NRF2, which in turn stimulate the expression of mtTFA. Importantly, PGC-1α has been shown to affect glucose metabolism. Under fed conditions, skeletal muscle is a major site of glucose disposal, taking up glucose from the plasma via GLUTs located in the cell membrane. In our study, the skeletal muscle tissue of sudachitin-treated mice showed increased transcription of GLUT4, a constitutively expressed gene encoding for an insulin-sensitive transporter protein. On the other hand, *in vitro* investigation in cultured myocytes has detected increased expression of GLUT1 and GLUT3, but not GLUT4, following incubation with sudachitin. One explanation for this apparent discrepancy might be that increased GLUT4 expression was secondary to the improvement in glucose metabolism caused by the decrease in weight gain *in vivo* (Figures 1 and 2), while sudachitin directly influences the expression of insulin-insensitive GLUTs in myocytes *in vitro*.

Interestingly, skeletal muscle-specific overexpression of PGC-1α was shown to be sufficient to delay many age-related disorders and extend the lifespan of mice [31]. The effects of sudachitin treatment seem to mimic those of caloric restriction and are similar to the reported effects of resveratrol. However, UCP1 and UCP3 expression were significantly increased in sudachitin-treated mice. Based on recent publications, it is possible that WAT was transformed into BAT-like tissue via PGC-1α activity [32], increasing tissue thermogenesis [30]. Clearly, further studies are required to fully understand the effect of sudachitin treatment and to elucidate the underlying mechanisms in these animal models.

The results of our current study suggest that sudachitin treatment increased serum adiponectin levels and increased Sirt1 expression, which activates PGC-1α in the skeletal muscle. Several reports suggest that AMPK and Sirt1 act together with PGC-1α, a key regulator of mitochondrial biogenesis, to regulate energy homeostasis in response to environmental and nutritional stimuli [33,34]. Rasbach et al. reported that overexpression of PGC-1α caused a shift toward increased oxidative types of skeletal muscle fibers [35]. Our *in vitro* data confirm that treatment with a moderate (30 μmol/L) dose of sudachitin induces mitochondrial biogenesis, and improves mitochondrial function. This dose also increased Sirt1 expression, suggesting that AMPK activation might be dependent upon Sirt1. Recently, Price et al. reported that moderate doses of resveratrol activate Sirt1, leading to a deacetylation of liver kinase B1 and AMPK activation, and that Sirt1 and AMPK do not function independently or linearly [36]. The dose of sudachitin used in our *in vivo* study was within physiologic levels because it is equivalent to the consumption of the peels of 1.5 small sudachi fruit per day in humans.

In this study, we examined the Sirt1-dependent activity of sudachitin in skeletal muscle, a tissue with very high energy demands that require highly efficient mitochondrial functioning. As a limitation to the presented work, it remains unclear whether Sirt1-independent effects observed here result from an accumulation of sudachitin or its metabolites in specific organs, or differences in the actions of sudachitin between tissues and cell types. It is also unclear how flavonoids, such as sudachitin, modulate Sirt1–PGC1α signaling and regulate specific transcription factors or whether these effects are mediated indirectly through a separate signaling pathway. Further studies are needed to gain more insight into the effects of sudachitin and its mechanisms in other tissues, particularly the liver and brain.

## Conclusions

In summary, our study is the first to demonstrate that sudachitin improves glucose and lipid metabolism by enhancing energy metabolism and mitochondrial biogenesis. Additionally, our *in vivo* and *in vitro* findings suggest that these effects are mediated by the Sirt1–AMPK–PGC-1α pathway. These observations indicate that sudachitin has potential for use in treating metabolic and age-related diseases.

## Competing interests

All authors (Rie Tsutsumi, Tomomi Yoshida, Yoshitaka Nii, Naoki Okahisa, Shinya Iwata, Masao Tsukayama, Rei Hashimoto, Yasuko Taniguchi, Hiroshi Sakaue, Toshio Hosaka, Emi Shuto, Tohru Sakai) have no conflicts of interest.

## Authors’ contribution

RT designed research, conducted the research, wrote the manuscript, and has responsibility for the final content; TY, RH and YT conducted research; HS, TH, ES and TS planed research and edited manuscript. YN, NO, SI and MT provided essential reagents and analyzed data. All authors have read and approved the final manuscript.

## Supplementary Material

Additional file 1: Table S1 Supplemental table Primer sequences. Click here for file
